# Enhancement of Non-photochemical Quenching as an Adaptive Strategy under Phosphorus Deprivation in the Dinoflagellate *Karlodinium veneficum*

**DOI:** 10.3389/fmicb.2017.00404

**Published:** 2017-03-15

**Authors:** Yudong Cui, Huan Zhang, Senjie Lin

**Affiliations:** ^1^State Key Laboratory of Marine Environmental Science and Marine Biodiversity and Xiamen City Key Laboratory of Urban Sea Ecological Conservation and Restoration, Xiamen UniversityXiamen, China; ^2^Department of Marine Sciences, University of Connecticut, GrotonCT, USA

**Keywords:** non-photochemical quenching, dinoflagellates, phosphorus deprivation, metabolic machinery reconfiguration, energy flow

## Abstract

Intensified water column stratification due to global warming has the potential to decrease nutrient availability while increasing excess light for the photosynthesis of phytoplankton in the euphotic zone, which together will increase the need for photoprotective strategies such as non-photochemical quenching (NPQ). We investigated whether NPQ is enhanced and how it is regulated molecularly under phosphorus (P) deprivation in the dinoflagellate *Karlodinium veneficum*. We grew *K. veneficum* under P-replete and P-depleted conditions, monitored their growth rates and chlorophyll fluorescence, and conducted gene expression and comparative proteomic analyses. The results were used to characterize NPQ modulation and associated gene expression dynamics under P deprivation. We found that NPQ in *K. veneficum* was elevated significantly under P deprivation. Accordingly, the abundances of three light-harvesting complex stress-related proteins increased under P-depleted condition. Besides, many proteins related to genetic information flow were down-regulated while many proteins related to energy production and conversion were up-regulated under P deprivation. Taken together, our results indicate that *K. veneficum* cells respond to P deprivation by reconfiguring the metabolic landscape and up-tuning NPQ to increase the capacity to dissipate excess light energy and maintain the fluency of energy flow, which provides a new perspective about what adaptive strategy dinoflagellates have evolved to cope with P deprivation.

## Introduction

Phytoplankton live in a constantly changing light environment affected by factors such as the strong solar radiation and fluctuant waves in the surface ocean, and they often absorb too much light which exceeds their photosynthetic capacity and would potentially cause photo-oxidative damage to the chloroplast (Anderson and Barber, 1996; Niyogi, 1999; Li et al., 2009). In response, these photosynthetic organisms have developed many photoprotective strategies to protect themselves from the damage of excess light, and one of the most important mechanisms is to dissipate the excessive excitation energy as heat through non-photochemical quenching of chlorophyll fluorescence (NPQ) (Horton and Ruban, 2005; Erickson et al., 2015; Goss and Lepetit, 2015). It was estimated that as high as 75% of the absorbed light energy could be eliminated by this thermal dissipation (Demmig-Adams et al., 1996; Niyogi, 1999). In the global ocean, about 60% of photons absorbed by marine phytoplankton are converted to heat (Lin H. et al., 2016).

Non-photochemical quenching consists of several components which are distinguished by different relaxation kinetics, among them the energy-dependent quenching (qE) is the most important and rapid part, which could be induced and relaxed within seconds to minutes and thus is especially important in coping with the frequent and rapid fluctuations of light intensities in the ambient environment (Horton and Hague, 1988; Müller et al., 2001; Zaks et al., 2013). The other NPQ components such as state-transition quenching (qT) and photoinhibitory quenching (qI) would relax within tens of minutes to hours (Müller et al., 2001; Erickson et al., 2015). In microalgae, qE relies on interconversion of specific pigments through xanthophyll cycle and the function of the light-harvesting complex stress-related family proteins LI818, which were known as LHCSR in green algae or LHCX in diatoms (Peers et al., 2009; Bailleul et al., 2010; Zhu and Green, 2010). Xanthophyll cycle in vascular plants, green and brown algae is composed of violaxanthin, antheraxanthin and zeaxanthin (VAZ cycle) while in dinoflagellates, diatoms and haptophytes it consists of diadinoxanthin and diatoxanthin (Dd-Dt cycle) (Masojídek et al., 2004; Goss and Jakob, 2010).

Excess light stress would be exacerbated especially when the photosynthetic organisms are exposed to various environmental stressors such as nutrient deprivation, which can lead to the reduction of the photosynthetic efficiency causing increase of excess excitation energy (Demmig-Adams and Adams, 1992; Wykoff and Grossman, 1998; Li et al., 2000). Thus, theoretically an increase of NPQ capacity is needed under these stress conditions (Demmig-Adams and Adams, 1992; Li et al., 2009). Previous researches have documented the transcriptional up-regulation of LHCSR genes under sulfur starvation and iron deficiency in the green alga *Chlamydomonas reinhardtii* (Zhang et al., 2004; Naumann et al., 2007). Enhanced NPQ capacity and elevated expression of specific LHCX genes and proteins under iron and nitrate starvation has also been reported in the diatom *Phaeodactylum tricornutum* (Taddei et al., 2016). However, NPQ capacity and LHCX gene expression were found to decrease under iron and copper limitation in the diatom *Thalassiosira pseudonana* (Zhu et al., 2010), and the amount of a LI818 related protein was also reduced significantly under lowered iron in another diatom *Cyclotella meneghiniana* (Beer et al., 2011), indicating that nutrient deprivation does not necessarily lead to NPQ induction in phytoplankton.

Phosphorus is an essential nutrient for the growth of marine phytoplankton, necessary for the synthesis of many essential P-contained biomolecules and plays an essential role in the regulatory of phosphorylation processes (Paytan and McLaughlin, 2007; Karl, 2014). However, the P directly available in the ocean, mainly in the form of orthophosphate, is often limited (Wu et al., 2000; Mills et al., 2004; Elser et al., 2007). Researchers have proposed the redirection of absorbed light energy through different components of NPQ during P starvation in *C. reinhardtii* (Wykoff and Grossman, 1998). The increase of LHCSR gene abundance under P deprivation and a possible role of the P-related transcription factor PSR1 in photoprotection has also been described in this species (Moseley et al., 2006). The rapid quenching of chlorophyll fluorescence through qE and qT during P*i* uptake were observed in P-starved green alga *Dunaliella tertiolecta* (Petrou et al., 2008). However, NPQ responses to P deprivation in dinoflagellates, an important group of eukaryotic phytoplankton in the marine ecosystem, which contribute significantly to the primary production, harmful algal blooms (HABs) and marine biotoxin production, remains to be explored. Furthermore, enhanced water column stratification due to recently increasing global warming suppresses the vertical mixing of water layers and thus reduces the nutrient supply to phytoplankton in the ocean’s upper layer (Behrenfeld et al., 2006; Coma et al., 2009; Hoegh-Guldberg and Bruno, 2010), predicting that the excess light stress induced by P deprivation in the future ocean will be worsened. Therefore, a better understanding on NPQ modulation under P deprivation in dinoflagellates will help us better understand how the algal group will adapt to the new environment in the future ocean.

*Karlodinium veneficum* is a cosmopolitan HAB-forming dinoflagellate species responsible for mass fish kills in many coastal areas of the world due to the production of karlotoxins which demonstrate hemolytic, cytotoxic, and ichthyotoxic properties (Peng et al., 2010, Place et al., 2012). In this study, NPQ estimation under contrasting P and light conditions was conducted in this species. We also studied expression dynamics of LHCX proteins through RT-qPCR and proteome analyses. Results showed that NPQ was elevated significantly not only when *K. veneficum* cells were exposed to high light, but also when they were P-deprived. A set of proteins was found differentially expressed between P-replete (+P) and P-depleted (–P) conditions, with three LHCX proteins and many other pigment proteins being up-regulated under the P-depleted condition. These results provide direct physiological evidence for enhanced NPQ in *K. veneficum* cells under P deprivation and the molecular mechanism of the response.

## Materials and Methods

### Algal Culture and Experimental Setup

*Karlodinium veneficum* strain CCMP2778 was originally isolated from coastal area off Longboat Key near Sarasota, Florida USA and provided by the Provasoli-Guillard National Center for Marine Algae and Microbiota (NCMA) in Boothbay Harbor, Maine, USA. In our laboratory, the culture was maintained in L1 medium (NCMA recipe) amended seawater (salinity, 28 PSU), which was filtered through 0.22-μm membranes and autoclaved. Cultures were grown at 20°C under a 14 h: 10 h light dark cycle with a photon flux of 100 ± 10 μmol photons m^-2^ s^-1^. To obtain the cultures under contrasting P conditions, the cultures were first grown in L1 medium until it reached the exponential growth stage, and were then inoculated into L1 and L1-P (same as L1 except that no phosphate was added) medium under the same light environment, both conditions were treated in triplicate. For the cultures under different light conditions, algal cells in the exponential growth stage were inoculated into new L1 medium to be cultured at 20°C under different light conditions (50, 300, and 600 μmol photons m^-2^ s^-1^) with previous diurnal cycle, each treated in triplicate. The cultures were acclimated to these light intensities for six generations before the measurements were made. The experimental cultures described above were grown in a volume of 300 mL in 500-mL flasks. Cell counts were carried out using a Sedgwick–Rafter counting chamber (Phycotech, St. Joseph, MI, USA). The concentration of dissolved inorganic phosphate (DIP) in the medium was measured using the molybdenum blue method (Timothy et al., 1984). About 1 × 10^6^ cells were collected from each culture at the selected time by centrifugation (5000 *g*, 10 min) and resuspended in 1 mL Trizol reagent (MRC, Cincinnati, OH, USA) and stored at –80°C for subsequent RNA extraction.

### NPQ Estimation by Chlorophyll Fluorescence Measurement

Chlorophyll fluorescence of *K. veneficum* cells was measured using a FIRe fluorometer system (Satlantic, Halifax, NS, Canada). The algal cell concentration was diluted to approximately 10, 000 cells per mL during the NPQ measurements to avoid self-shading effect. The high luminosity blue light (maximum emission 455 nm, 60 nm bandwidth) in FIRe was used to excite chlorophyll fluorescence. *K. veneficum* cells were sampled at the 8th hour of the light cycle and were then dark adapted for 30 min at 20°C before measurement. NPQ was calculated as (Fm-Fm’)/Fm’ and the maximum quantum efficiency of PSII photochemistry Fv/Fm = (Fm–F_0_)/Fm (Maxwell and Johnson, 2000; Baker, 2008), where F_0_ is the minimal fluorescence obtained in the presence of the measuring light; Fm is the maximum fluorescence of dark-adapted algal cells measured during a very short and strong single turnover flash (STF); and Fm’ is the maximum fluorescence measured after the cultures were exposed to a continual actinic light (PAR, photosynthetically active radiation, wavelength range from 400∼700 nm) using the actinic light source (ALS) through the manual PAR acquisition or PAR stepping acquisition of FIRe. NPQ induction and relaxation kinetics under contrasting light and P conditions were observed through the manual PAR acquisition, algal cells after dark-adaption were exposed to actinic light for 10 min and then the actinic light was turned off for another 10 min, Fm’ were measured at the end of each minute during this process and NPQ was calculated accordingly. The algal cells cultured under 100 μmol photons m^-2^ s^-1^ were sampled for NPQ estimation under contrasting actinic light intensities. To measure the induction and relaxation of NPQ under contrasting P conditions, the algal cells were sampled at the 10th day upon inoculation to +P and –P conditions. The maximal NPQ capacity of the algal cells under +P and –P condition was also measured using the PAR stepping acquisition, in which the PAR intensity increased by 50 every 30 s from 0 to 700 μmol photons m^-2^ s^-1^.

### Identification and RACE (Rapid Amplification of cDNA Ends) of Genes Related to Photoprotection

We investigated our annotated dinoflagellate-specific spliced leader (DinoSL)-based *K. veneficum* cDNA database (Lin et al., unpublished, as briefly reported in Cui et al., 2016) for genes potentially related to NPQ. The sequences acquired were further confirmed by blastp against NCBI GenBank database. To obtain the full-length cDNA of these genes, we extracted RNA from *K. veneficum* cells as previously reported (Lin et al., 2010). Specific primers (Supplementary Table S1) were designed for both 3′- and 5′- RACE based on the partial sequences identified from the above-mentioned transcriptome dataset. The 21-bp highly conserved DinoSL was used as the 5′ forward primer for the 5′-RACE (Zhang et al., 2007; Lin et al., 2010). The amplicons were cloned into T-vectors and sequenced through Sanger sequencing.

### Expression Dynamics of NPQ-Related Genes Measured Using RT-qPCR

Specific primers (Supplementary Table S1) were designed for RT-qPCR to examine the differential expression of the photoprotection genes identified in this work under different P and light conditions. *Calmodulin* (calcium-modulated protein; KM275627) was used as the reference gene because of its relative stable expression previously reported in some other dinoflagellates (Rosic et al., 2011; Shi et al., 2013). For standard curves, a purified PCR product for each gene was prepared in ten-fold dilution series (10^3^–10^7^ copies per μL). RT-qPCR was performed using Bio-Rad iQ SYBR Green Supermix Kit (Bio-Rad Laboratories, Hercules, CA, USA) with all the reactions set up in triplicate for each gene. Relative transcript levels of these genes were calculated in two ways to facilitate comparison: normalized to the amount of total RNA equivalent to the amount of cDNA used in each reaction, and to the expression levels of the reference gene *calmodulin*.

### Comparative Proteomic Analysis

We carried out iTRAQ (isobaric tags for relative and absolute quantitation) analysis to identify the differentially expressed proteins in *K. veneficum* collected from +P (L1) and -P (L1-P) conditions. Cultures under contrasting P conditions were obtained as described above and grown in a volume of 1 L in 2-L flasks. After inoculation into different conditions, the +P cells were sampled at the 3rd day, and the –P cells were sampled at the 9th day, each in duplicate. About 3 × 10^7^ algal cells were harvested from each culture and protein was extracted as previously reported (Wang et al., 2013). Total protein was quantified through Bradford protein assay and 100 μg from each sample was used for iTRAQ labeling. Samples were labeled with the iTRAQ tags and SCX-fractionated with a LC-20AB HPLC pump system (Shimadzu, Kyoto, Japan). Liquid chromatography electrospray ionization tandem mass spectrometry (LC-ESI-MS/MS) analysis was then performed based on a TripleTOF 5600 System (AB SCIEX, Concord, ON, USA), followed by protein identification through Mascot search engine (Matrix Science, London, UK; version 2.3.02) against the above-mentioned DinoSL-based *K. veneficum* cDNA database. To reduce the probability of false peptide identification, only peptides at the 95% confidence interval by a Mascot probability analysis greater than “identity” were accepted, and each confident protein identification was represented by at least one unique peptide.

The abundance of a protein was quantified only when it was represented by at least two unique peptides in our proteomic data. The quantitative protein ratios were weighted and normalized by the median ratio in Mascot. The criteria as fold changes >1.2 and *p*-values < 0.05 was adopted to depict significantly differentially expressed proteins. Functional annotations of the proteins were conducted using Blast2GO program against NCBI non-redundant (nr) protein database and Uniprot database^1^. The KEGG database^2^ and the Clusters of Orthologous Groups (COG) database^3^ were used to classify and group these identified proteins.

### Statistical Analysis

Analysis of variance (ANOVA) was carried out using PASW Statistics 18 software package to evaluate the statistical significance of the differences between contrasting light and P conditions. Data shown in the figures are means with standard deviation calculated from different replicates.

## Results

### NPQ Induction under High Light Stress

**FIGURE 1:**
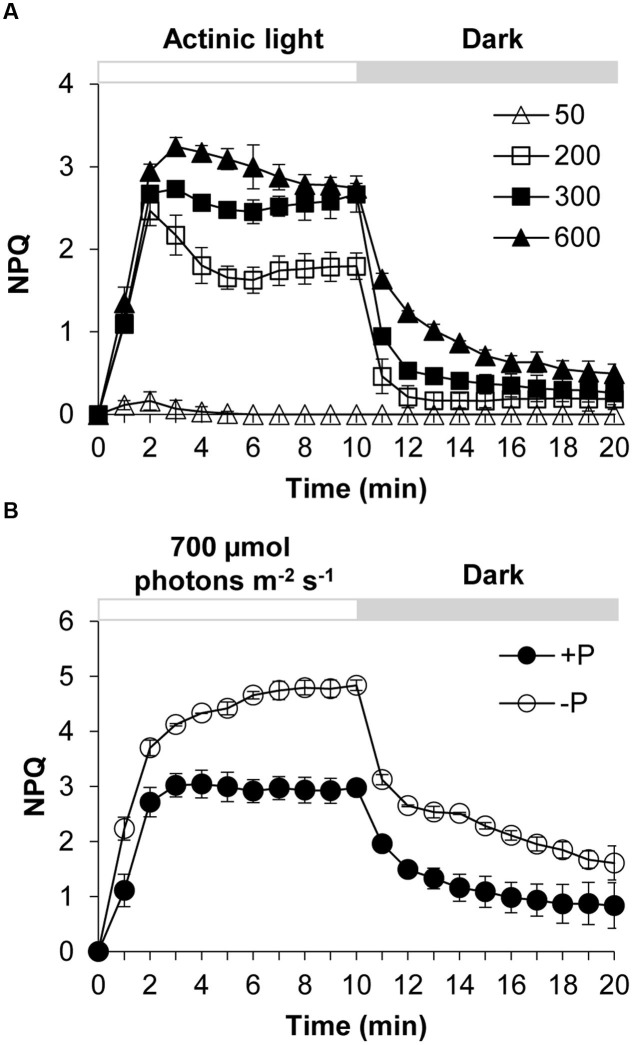
**Dynamics of NPQ (non-photochemical quenching) under contrasting light and P (phosphorus) conditions in *Karlodinium veneficum*.** The dark-adapted algal cells were exposed to actinic light for 10 min to induce the NPQ and then the actinic light was turned off for another 10 min. Fm’ was measured at the end of each minute. **(A)** Induction and relaxation of NPQ of *K. veneficum* cells exposed to different actinic light intensities. Open triangles, actinic light of 50 μmol photons m^-2^ s^-1^; open squares, actinic light of 200 μmol photons m^-2^ s^-1^; closed squares, actinic light of 300 μmol photons m^-2^ s^-1^; closed triangles, actinic light of 600 μmol photons m^-2^ s^-1^. **(B)** Induction and relaxation of NPQ of *K. veneficum* cells grown under +P and –P conditions and an actinic light intensity of 700 μmol photons m^-2^ s^-1^. Closed circles, P-replete condition; open circles, P-depleted condition. Data shown are means ± SD (error bars) from the triplicated measurements.

*Karlodinium veneficum* cells after dark-adaption were exposed to different light intensities of actinic light using FIRe to observe the induction and relaxation of NPQ (**Figure 1A**). Under actinic light of 50 μmol photons m^-2^ s^-1^, the NPQ was induced briefly at the first three minutes and then the NPQ returned to zero, indicating that under this light intensity the algal cells did not need NPQ to dissipate the excess light. The increase of NPQ in the beginning was due to the sudden shift of dark-adapted cells to the light. When the dark-adapted algal cells were exposed to actinic light of 200, 300, and 600 μmol photons m^-2^ s^-1^, NPQ was induced quickly and significantly. After light was turned off at the 10th minute, NPQ relaxed quickly but not completely in several minutes. During the first two to three minutes of the light phase, an abrupt increase in NPQ was observed from the dark to light transition. Subsequently the NPQ showed slightly different fluctuations under the three different light conditions. Under 200 and 300 μmol photons m^-2^ s^-1^, the NPQ value decreased to a minimum and then increased to a relatively steady state before a sharp decline occurred at the 10th minute. Under 600 μmol photons m^-2^ s^-1^, the NPQ decreased gradually until it reached a relatively steady value.

### Increase of NPQ Capacity under P-Depleted Condition

The NPQ induction and relaxation of *K. veneficum* cells under +P and –P conditions were studied through a continual exposure to 700 μmol photons m^-2^ s^-1^ for 10 min followed by dark treatment for 10 min (**Figure 1B**). The results showed that NPQ under P-depleted condition was induced more quickly and the values were generally higher compared to that under P-replete condition. Upon switch to dark, NPQ relaxed quickly while it still maintained at a higher level for the P-depleted cells compared to the P-replete cells.

**FIGURE 2:**
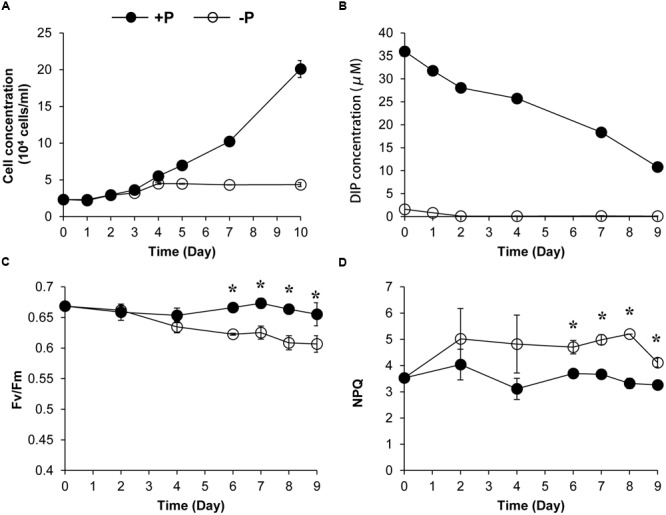
**Growth curves **(A)**, dissolved inorganic phosphate (DIP) concentration change **(B)**, Fv/Fm **(C)**, and NPQ capacity **(D)** of *K. veneficum* cells grown under +P and –P conditions.** Closed circles, P-replete condition; open circles, P-depleted condition. Data shown are means ± SD (error bars) from the triplicated cultures. Asterisks represent that significant differences were detected (*p* < 0.05) between +P and –P conditions.

We also conducted a 10-day experiment to further estimate the NPQ capacity and the Fv/Fm under the two P conditions. Algal growth rate under +P condition was higher than that under the –P condition (**Figure 2A**). The DIP depletion and the cessation of the population growth under the –P condition indicated that the cultures were experiencing P deprivation from the fourth day of the experiment (**Figures 2A,B**). Fluorescence measurement showed that the Fv/Fm decreased over time under the -P condition while that in the +P cultures kept at relatively stable and higher levels (**Figure 2C**), indicating that P deprivation led to a lower photochemical efficiency. In accordance, the NPQ capacity of the P-depleted cells was significantly higher than that of P-replete cells (**Figure 2D**).

### Transcriptional Dynamics of Photoprotection Genes under Different P Conditions

**FIGURE 3:**
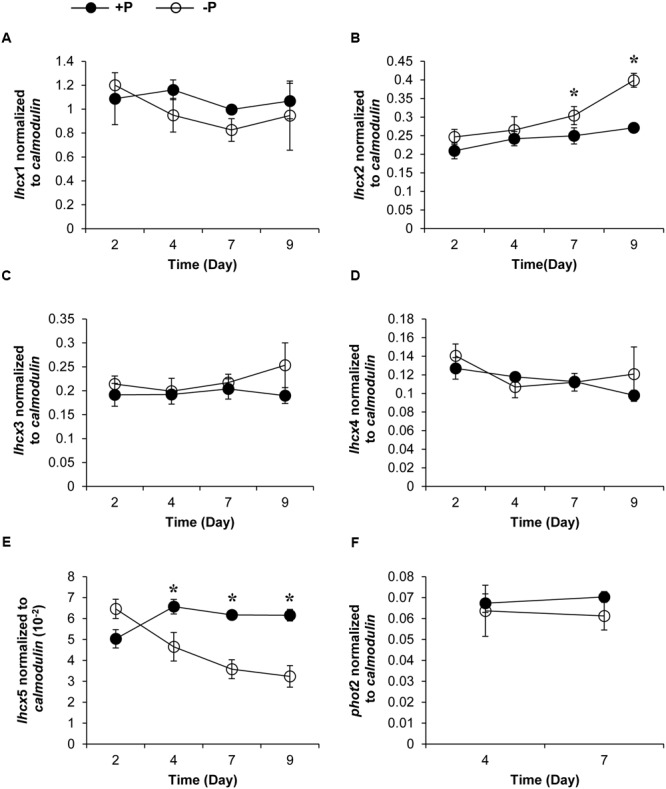
**Transcript abundances of *lhcx*1 **(A)**, *lhcx*2 **(B)**, *lhcx*3 **(C)**, *lhcx*4 **(D)**, *lhcx*5 **(E)**, and *phot*2 **(F)** genes normalized to *calmodulin* in *K. veneficum* cells grown under +P and –P conditions** (the same batch cultures as **Figure 2**). Closed circles, P-replete condition; open circles, P-depleted condition. Shown are means ± SD (error bars) from the triplicated cultures. Asterisks represent that significant differences were detected (*p* < 0.05) between +P and –P conditions.

Genes encoding five LHCX proteins (Genebank No. KX524133 to KX524137) and a homolog of Phototropin-2 (PHOT2, KX524138), which is involved in chloroplast avoidance movement in plants (Kasahara et al., 2002), were identified in the cDNA library of *K. veneficum* (**Table 1**). With specific primers designed from the partial sequences obtained, our RACE yielded cDNAs containing the complete open reading frame (ORF), with existence of DinoSL at the 5′ UTR of these gene transcripts confirming their dinoflagellate origin. RT-qPCR was conducted to study their transcript abundances. The results showed that the transcriptional regulation of the *lhcx* and the *phot*2 genes was quite limited in response to different P conditions (**Figure 3**). Transcript abundances of *lhcx*1, 3, 4 and *phot*2 did not show significant differences between +P and –P conditions. However, *lhcx*2 showed a significant up-regulation under –P condition (**Figure 3B**). Moreover, *lhcx*5 was remarkably down-regulated under the P-depleted condition compared to P-replete condition (**Figure 3E**).

**Table 1:** Identification of proteins related to NPQ (non-photochemical quenching) in *Karlodinium veneficum* based on RACE and iTRAQ analysis.

Sequence ID	DinoSL	ORF length (bp)	Deduced protein length (aa)	Annotation source	Description	*E*-value	Identity (%)
Karve LHCX 1	√	783	261	NCBI Blastp	fucoxanthin chlorophyll a/c LI818 clade [*Chrysochromulina* sp. CCMP291]	5.00E-25	42
Karve LHCX 2	√	831	277	NCBI Blastp	plastid light harvesting protein LI818 [*Dinophysis acuminata*]	3.00E-30	40
Karve LHCX 3	√	753	251	NCBI Blastp	fucoxanthin chlorophyll a/c protein, LI818 clade [*Thalassiosira pseudonana* CCMP1335]	6.00E-29	40
Karve LHCX 4	√	747	249	NCBI Blastp	plastid light harvesting protein LI818 [*Dinophysis acuminata*]	2.00E-52	54
Karve LHCX 5	√	786	262	NCBI Blastp	fucoxanthin chlorophyll a/c protein, LI818 clade [*Thalassiosira pseudonana* CCMP1335]	1.00E-26	37
Karve PHOT2	√	825	275	Uniprot_ Swissprot	Phototropin-2[*Arabidopsis thaliana*]	1.00E-11	60
Karve VDE1	√	1326	442	NCBI Blastx	violaxanthin de-epoxidase [*Chrysochromulina* sp. CCMP291]	7.00E-136	63
Karve VDE2	√	1203	401	NCBI Blastx	violaxanthin de-epoxidase [*Chrysochromulina* sp. CCMP291]	9.00E-165	73
Karve ZEP	√	1740	580	NCBI Blastx	zeaxanthin epoxidase [*Chrysochromulina* sp. CCMP291]	0.00	61

### Transcriptional Dynamics of Photoprotection Genes under Different Light Conditions

**FIGURE 4:**
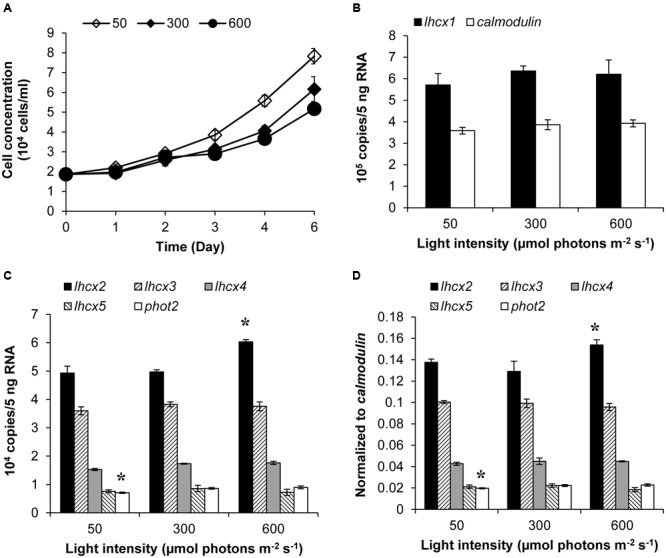
**Growth curves **(A)**, transcript abundances of *lhcx*1 compared to *calmodulin***(B)**, transcript abundances of *lhcx*2, 3, 4, 5, and *phot*2 normalized to 5 ng RNA **(C)** and normalized to *calmodulin***(D)** of *K. veneficum* cells grown under different light intensities.** The samples for gene expression analysis were collected on the 6th day. Shown are means ± SD (error bars) from the triplicated cultures. Asterisks represent that the condition is significantly different from the other two conditions.

Transcript abundances of LHCX proteins and PHOT2 in *K. veneficum* cells cultured under different light conditions were also studied through RT-qPCR. The growth rate of cultures under 50, 300, and 600 μmol photons m^-2^ s^-1^ was 0.239, 0.2, and 0.17, respectively, indicating that under the three light intensities employed in this study, the higher the light intensity used, the lower growth rate the cultures achieved (**Figure 4A**). The transcript abundance of *lhcx*1 was very high, even higher than *calmodulin*, the reference gene used in this study; however, the transcript level was similar under the three light conditions (**Figure 4B**). Similarly, *lhcx*3, 4 and 5 did not show a significant differential expression under the three light conditions (**Figures 4C,D**). In contrast, *lhcx*2 exhibited changes in transcript abundance, higher under 600 μmol photons m^-2^ s^-1^ than under 50 and 300 μmol photons m^-2^ s^-1^ with the latter two conditions producing no significant difference. *Phot*2 also showed a transcriptional regulation in response to different light conditions but the pattern was different from *lhcx*2. The transcript abundance of *phot*2 was higher at the light intensities of 300 and 600 μmol photons m^-2^ s^-1^ than at 50 μmol photons m^-2^ s^-1^, albeit at a small magnitude, but the former two light conditions did not elicit significant difference.

### Comparative Proteomic Analysis under +P and –P Conditions and Identification of Proteins Related to Photoprotection in *K. veneficum*

**FIGURE 5:**
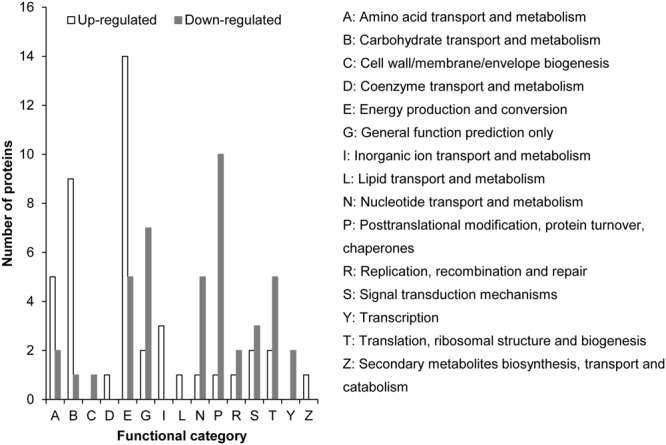
**Distribution of up-regulated (empty bar) and down-regulated (solid bar) proteins under –P condition compared to +P condition based on iTRAQ comparative proteomic analysis in *K. veneficum*.** The proteins for which we could not identify a COG functional category were excluded in this analysis.

The iTRAQ proteomic analysis identified 4, 922 proteins for *K. veneficum* grown under +P and –P conditions (Supplementary Figure S1); 82 of them were found to be up-regulated under the –P condition (Supplementary Table S2), with 43 being classified into different COG functional categories, mainly carbohydrate metabolism, energy production and amino acid metabolism (**Figure 5**). Among these up-regulated gene categories there were two inorganic pyrophosphatases, which catalyze the hydrolysis of pyrophosphate into phosphate. Also identified were a glycerol-3-phosphate dehydrogenase and a putative sterol carrier protein, which might be involved in lipid metabolism. Three proteins were related to inorganic ion transport and sulfur metabolism. One photosystem II protein and two mitochondrial tricarboxylate transporters were also identified among the up-regulated proteins.

Totally 136 proteins were down-regulated under the –P condition and 41 of them were classified into 11 COG functional categories (**Figure 5** and Supplementary Table S3). Most of these proteins are related to nucleic acid and protein synthesis (e.g., involved in nucleotide transport and metabolism, DNA replication, transcription, translation, and posttranslational modification, **Figure 5**). Three proteins in signal transduction pathways, including two Ca^2+^-binding proteins, were identified. A predicted histidines or aspartates domain phosphohydrolase was also down-regulated under the –P condition (Supplementary Table S3). From the 93 down-regulated proteins that were not grouped into COG functional categories, a thylakoid luminal protein, a polymerase, a peptidase, a deoxyribonuclease II, a cold shock protein, two RNA-binding proteins, two cathepsins and four enzymes related to amino acid metabolism were identified (Supplementary Table S3). Most of the other proteins were annotated as putative uncharacterized proteins and predicted proteins. Fifty-four other down-regulated proteins under P stress had no matches in the database, potentially novel proteins responding to P stress.

**FIGURE 6:**
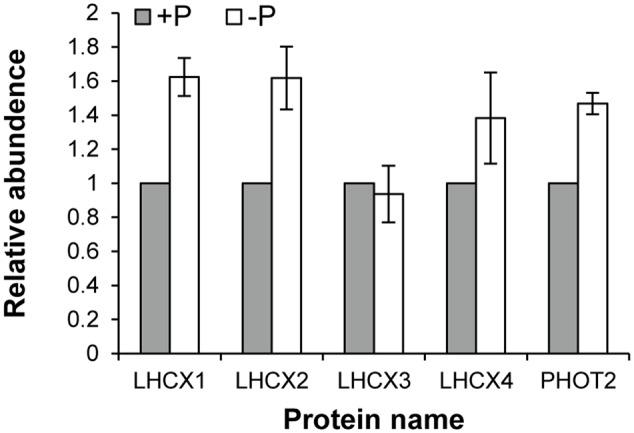
**Relative abundances of LHCX and PHOT2 proteins in *K. veneficum* grown under –P (empty bar) versus +P (solid bar) conditions based on the iTRAQ comparative proteomic analysis.** The expression levels of each protein under +P condition were set as one.

Seventy-two light harvesting protein complexes (LHCs) were identified and five of them were annotated as stress-related chlorophyll *a*–*b* binding proteins LI818 (Savard et al., 1996; Richard et al., 2000; Peers et al., 2009), denoted as LHCX proteins in this study (**Table 1**). Totally 27 of the LHCs were up-regulated under the –P condition (Supplementary Table S2), these included three LHCX proteins, LHCX1, LHCX2, and LHCX4 (**Figure 6**). LHCX5 was excluded from the comparative proteomic analysis because its abundance was too low. PHOT2 was identified in the *K. veneficum* proteome and its abundance was also found to be higher under the –P condition (**Figure 6**). Besides, two VDE proteins (KX524139 and KX524140) and one ZEP protein (KX524141) were also identified in the proteome and cDNA library of *K. veneficum* (**Table 1**).

## Discussion

### NPQ in *K. veneficum*

Phytoplankton live in surface water and often face excess light, and thus have developed many strategies to protect them from photo-oxidative damage caused by excess light energy (Niyogi, 1999; Erickson et al., 2015). While recent study about NPQ in dinoflagellates mainly focus on the symbiotic species of corals, NPQ mechanism in HAB-forming dinoflagellate species, which play an ecologically important role in the marine ecosystem, has rarely been explored (Goss and Lepetit, 2015). From the induction kinetics of NPQ observed in the present study, it is evident that transfer from darkness to light, even low light, can lead to a brief induction of NPQ in *K. veneficum* cells. Within the range used in this study (50–600 μmol photons m^-2^ s^-1^), NPQ increased with the intensity of ambient light. Reduced growth rate of the *K. veneficum* cultures was found under high light condition in this study (**Figure 4A**), indicating that excess light caused photoinhibition to this species. The induction of NPQ under high light and its relaxation in the dark shows that NPQ is an important mechanism for *K. veneficum* to cope with absorbed excess light energy. Our results showed that the induction and relaxation of the NPQ in *K. veneficum* was very rapid, indicating that qE, which enables the organism to cope with the frequently and rapidly changing light field in the coastal marine ecosystem, is a major constituent of NPQ in this species, as is the case for most plants and algae (Müller et al., 2001; Erickson et al., 2015; Goss and Lepetit, 2015) Moreover, from our *K. veneficum* proteomic dataset, we detected the LHCX proteins, which have been confirmed to play a vital part in the qE of many microalgal groups (Peers et al., 2009; Bailleul et al., 2010; Goss and Lepetit, 2015). NPQ did not relax completely in ten minutes under dark environment, especially in the P-deprived condition, suggesting that other components of NPQ such as qT and qI, which needs longer time to relax, were also induced, as has been described in other algae (Wykoff and Grossman, 1998; Zhu and Green, 2010). It has also been proposed that diatoxanthin could also contribute to the sustained part of NPQ (Lavaud and Lepetit, 2013).

Earlier pigment composition analysis revealed presence of violaxanthin but absence of antheraxanthin and zeaxanthin in *K. veneficum* CCMP2778, while the diadinoxanthin and diatoxanthin were very abundant in this strain (Bachvaroff et al., 2009), suggesting that Dd-Dt cycle is the main xanthophyll cycle in this species. This corresponds to previous findings that xanthophyll cycle in other groups of dinoflagellates is also made of the Dd-Dt cycle (Demers, 1991; Ambarsari et al., 1997). Violaxanthin in this species could be the precursors of diadinoxanthin, diatoxanthin and fucoxanthin, as previously reported in other algae containing Dd-Dt cycle (Lohr and Wilhelm, 1999; Goss and Jakob, 2010). The VAZ cycle is catalyzed by violaxanthin de-epoxidase (VDE) and zeaxanthin epoxidase (ZEP) while the Dd–Dt cycle depends on diadinoxanthin de-epoxidase (DDE) and diatoxanthin epoxidase (DEP) (Goss and Jakob, 2010; Goss and Lepetit, 2015). DDE and DEP were detected from our *K. veneficum* proteome but were annotated as VDE and ZEP (**Table 1**), as in the case of the diatoms *P. tricornutum* and *T. pseudonana* (Goss and Lepetit, 2015), because VDE versus DDE and ZEP versus DEP have high sequence identities (Coesel et al., 2008). VDE and DDE would differ in optimal activation pH, and ZEP and DEP differ in the regulation of enzyme activity (Jakob et al., 2001; Goss and Jakob, 2010). The exact structural and functional nature of the xanthophyll cycle in the dinoflagellates requires further studies.

### Transcriptional and Translational Regulation of Photoprotection Proteins

From the RT-qPCR results, four of the five identified *lhcx* genes did not show transcriptional regulation under different light intensities, although the growth rate and the induced NPQ of the algal cells under the three light conditions were very different (**Figure 4**). *Lhcx*2 and *phot*2 were differentially expressed at the transcriptional level according to ANOVA; however, the fold change is rather limited. The limited transcriptional regulation of the photoprotection genes in *K. veneficum* cells was also found when they were cultured under different P conditions (**Figure 3**). Three *lhcx* genes (*lhcx*1, 3, and 4) as well as *phot*2 did not show transcriptional regulation between P-replete and P-depleted conditions. Interestingly, although both were *lhcx* genes, *lhcx*2 was up-regulated while *lhcx*5 was down-regulated under the P-depleted condition compared to the P-replete condition, indicating that different LHCX genes might respond differently to different environmental stresses. Similar results have also been observed in diatoms under different light and nutrient stresses, reflecting functional diversification of the LHCX gene family in the microalgal groups which enable them to adapt to different ecological niches in the ocean (Bailleul et al., 2010; Zhu et al., 2010; Taddei et al., 2016).

On the other hand, the iTRAQ analysis showed that the expression of LHCX1, 2, 4 and PHOT2 proteins were up-regulated under the P-depleted condition, indicating that the regulation of most of the LHCX proteins (except LHCX2) and PHOT2 rest at different levels. It seems that the regulation of the LHCX proteins and PHOT2 in *K. veneficum* lies mainly in the translational level. The nonsynchronous regulation of these proteins at the transcriptional and translational levels might be because the transcriptional regulation of genes in *K. veneficum* was quite limited, as has been observed in many other dinoflagellates (Lin, 2011).

### NPQ Enhancement as an Adaptive Mechanism to Cope with P Deprivation in *K. veneficum*

**FIGURE 7:**
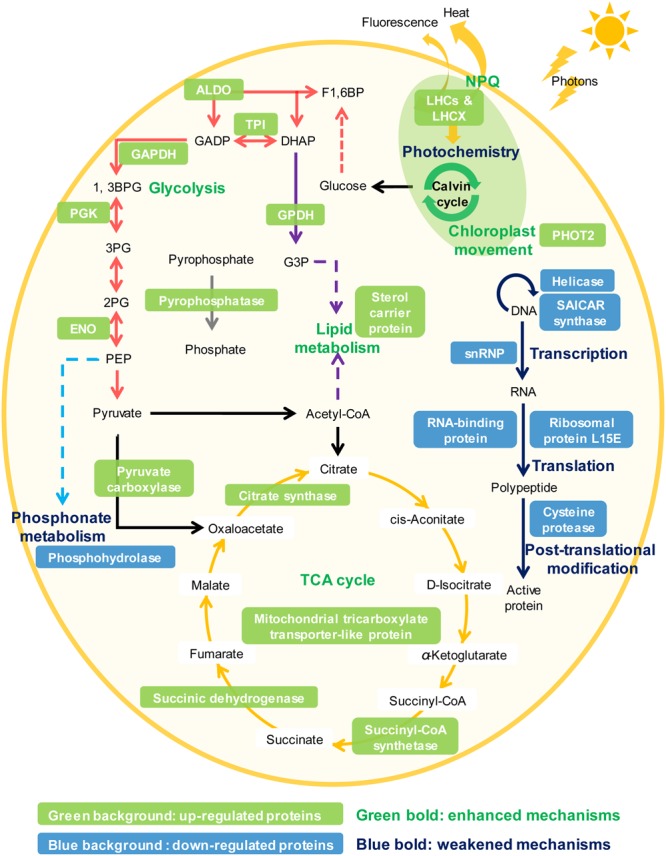
**Schematic representation of the NPQ enhancement and the metabolic machinery reconfiguration in *K. veneficum* under P deprivation inferred from the iTRAQ comparative proteomic analysis.** Glycolysis (red arrows), tricarboxylic acid (TCA) cycle (orange arrows), lipid metabolism (purple arrows), pyrophosphate hydrolysis (gray arrows), NPQ and chloroplast avoidance movement were strengthened while photochemistry, genetic information flow (e.g., DNA replication, transcription, translation, and post-translation, blue arrows) and phosphonate metabolism (light blue arrows) were weakened under P deprivation in *K. veneficum*. Representative up-regulated or down-regulated proteins under P deprivation are indicated in white font with a green or blue background, respectively. LHCs, light harvesting protein complexes; LHCX, light-harvesting complex stress-related family proteins; PHOT2, Phototropin-2; ALDO, fructose-bisphosphate aldolase; TPI, triosephosphate isomerase; GAPDH, glyceraldehyde phosphate dehydrogenase; PGK, phosphoglycerate kinase, ENO: enolase; GPDH, Glycerol-3-phosphate dehydrogenase; snRNP, small nuclear ribonucleoprotein.

Phytoplankton have evolved many strategies to cope with P deprivation, including reducing the cellular demand of P and enhancing the ability to utilize other P sources such as dissolved organic phosphorus (Dyhrman et al., 2007; Van Mooy et al., 2009; Lin S. et al., 2016). Our results showed that although *K. veneficum* experienced growth inhibition under P deprivation, it could still maintain a stable population for an extended period of time (**Figure 2**). Accordingly, the comparative proteomic analysis reveals significant reconfiguration of the metabolic machinery in *K. veneficum* under P deprivation (**Figure 7**). Many proteins involved in the genetic information flow (e.g., DNA replication, transcription, translation and post-translation) were down-regulated to reduce the demand of P as protein synthesis is one of the major P sinks (Lin S. et al., 2016). Similarly, phosphonate metabolism was slowed down under P-depleted condition (Cui et al., 2016). However, *K. veneficum* cells maintained and even strengthened the functions related to energy production and processes demanding less P such as glycolysis pathway, tricarboxylic acid (TCA) cycle and lipid metabolism. Meanwhile, pyrophosphatases were up-regulated, which could hydrolyze pyrophosphate to release phosphate. Furthermore, the abundances of over a third of the LHCs were up-regulated under P-depleted condition. The metabolic machinery reconfiguration is consistent with the proposal that phytoplankton could increase the proportion of resource acquisition machinery such as P-poor proteins and pigments and decrease the production of growth machinery such as ribosomal RNA when resources are scarce (Klausmeier et al., 2004; Arrigo, 2005). Similar proteomic landscape changes such as the elevation of the ability to scavenge or economize P, increase of the LHC abundances, adjustment of the glycolysis pathway and down-regulation of protein synthesis under P deprivation have also been documented in the diatom *T. pseudonana* and the pelagophyte *Aureococcus anophagefferens* (Wurch et al., 2011; Dyhrman et al., 2012).

It is interesting to observe elevation in NPQ and up-regulation of NPQ-related and other photoprotective proteins in *K. veneficum* in response to P deprivation. The identification and the up-regulation of PHOT2 under P-depleted condition suggest that *K. veneficum* can potentially perform chloroplast avoidance movement to reduce the absorption of photons by the chloroplast. The higher NPQ measured in the P-depleted condition indicates that the algal cells could enhance their NPQ activity under P stress to dissipate the excess light stress and protect them from the potential photo-oxidative damage. This is the first documentation of this phenomenon in dinoflagellates, to the best of our knowledge. Our proteomic and transcriptional analyses discussed above, including the up-regulation of LHCX1, 2, and 4, have provided molecular evidence for the enhanced NPQ under P deprivation in *K. veneficum*.

The increase in the abundances of many light-harvesting proteins and the enhanced function of metabolic machineries related to energy production and conversion such as glycolysis, TCA cycle and pyrophosphate hydrolysis in the P-deprived cells indicate that the acquisition of light energy and the downstream energy flow were enhanced in the P-deprived cells (**Figure 7**). We suggest that the energy flow was accelerated in the P-deprived cells to increase the recycling rate of P-containing compounds such as ATP and NADPH to compensate for the very low external supply of P. Besides, ATPs generated from these metabolic processes are supposed to supply the energy needed for P*i* acquisition, as the uptake of low concentration P*i* and the utilization of DOPs from ambient environment by the P-deprived cells require energy (Petrou et al., 2008; Lin S. et al., 2016).

Despite the increased absorption of light energy, the algal cultures in our study exhibited a compromised photosynthetic efficiency when P was deprived, which is similar to the case of another dinoflagellate *Amphidinium carterae* (Li et al., 2016). The decreased photochemical efficiency would aggravate excess light stress (Wykoff and Grossman, 1998). Under this condition, elevated thermal dissipation through NPQ could protect the photosynthetic apparatus from photodamage and maintain the fluency of the energy flow. As photosynthesis is the basis of energy acquisition in algae, protection of the photosynthetic apparatus provided by the enhanced NPQ would be critical to the vulnerable algal cells suffering from P deprivation in a complex and changing light environment. Thus, NPQ plays an important role in the modulation of light energy and keeps the balance of the energy budget in the P-deprived algal cells. There could also be some interactions between light intensities and P-deprivation related to the regulation of NPQ capacity, which needs further work to be explored in the future.

Taken together, the results from this study suggest that NPQ functions are not only a protection from high light condition, but can also be an important adaptive mechanism for algal cells to cope with P deprivation. It gives flexibility to the P-deprived algal cells which need to acquire more energy with a lower photochemical efficiency to fuel the P acquisition processes and compensate for the P deprivation. This mechanism together with other photoprotective strategies could maintain the operation of photosynthesis and downstream functions related to energy flow and conversion and thus serve as an essential survival strategy for the dinoflagellate under P deprivation.

## Concluding Remarks

In this study, using an integrative approach, we discovered that the dinoflagellate *K. veneficum* could reconfigure their cellular metabolic machinery and regulate expression dynamics of specific proteins related to NPQ to cope with excess light stress and balance the energy budget under P deprivation. In particular, this species up-regulates many proteins related to light modulation such as LHCX proteins and PHOT2 under the P-depleted condition. Accordingly, NPQ function was also elevated significantly when *K. veneficum* cells were P stressed, suggesting that this could be an important adaptive strategy for this species to cope with P deprivation. The multi-faceted machinery of photoprotection may confer *K. veneficum* a competitive advantage in facing global warming that will exacerbate excess light energy and nutrient deprivation. Further work is needed to address how the photoprotective machinery evolved, and whether the deprivation of other nutrients such as N and Fe will also promote NPQ capacity in this and other dinoflagellates.

## Author Contributions

YC and SL designed the research and wrote the paper; YC performed the laboratory work and data analysis; HZ made the *K. veneficum* cDNA library used for proteome data mapping.

## Conflict of Interest Statement

The authors declare that the research was conducted in the absence of any commercial or financial relationships that could be construed as a potential conflict of interest.

## Footnotes

1 http://www.uniprot.org/

2 http://www.genome.jp/kegg/

3 http://www.ncbi.nlm.nih.gov/COG/
