# Supplementary material for: Enhancement of Non-photochemical Quenching as an Adaptive Strategy under Phosphorus Deprivation in the Dinoflagellate Karlodinium veneficum
Source: Front Microbiol. 2017 Mar 15;8:404. doi: 10.3389/fmicb.2017.00404 (PMC5350143; doi:10.3389/fmicb.2017.00404)
Supplement: Supplementary file 1 [file Table_1.PDF]

Supplementary Table S1 Primers and their annealing temperatures used in this study.

Primer name	Primer sequence (5'-3')	Application	Annealing temperature/℃
DinoSL (F)	CCGTAGCCATTTTGGCTCAAG	5'-RACE	56
454BT7 (R)	CTATGCGCCTTGCCAGCCCGCTCAGTAATA CGACTCACTATAGGGAG	3'-RACE	56
454BT7 dT	GAGACTATGCGCCTTGCCAGCCCGCTCAGT AATACGACTCACTATAGGGAG(T)16VN	cDNA synthesis	42
Oligo-dT	$(T)_{16}$VN	cDNA synthesis	42
*lhcx*1 F0	TTCGTGAGTTGGCTGCGTAG	Coding region amplification	56
*lhcx*1 R0	CCAATGAGCTAATCACCGAAGG	Coding region amplification	56
*lhcx*2 F1	CGTACACTGCGCTCGCGTTGATT	Coding region amplification	56
*lhcx*2 R2	AGCACGAACCAAGAGCATGATGAGAA	Coding region amplification	56
*lhcx*3 F1	GCAATGCGGACTGTCGTTCCTGTT	Coding region amplification	56
*lhcx*3 R1	TGCTCTTACGCACGATTCAAGCCAAT	Coding region amplification	56
*lhcx*4 F1	AGCGATTAGTTGAATCCTGGCTATGT	Coding region amplification	56
*lhcx*4 R1	GCATGGCAATCTGCTGCTCAAGA	Coding region amplification	56
*lhcx*5 F1	TTCGTGAGTTGGCTGCGTAGAA	Coding region amplification	56
*lhcx*5 R2	TCTGCACACGCCTTGTCACAATGAG	Coding region amplification	56
*phot*2 F2	AGGTTGACGATGTTGAAGAGGAAGG	3' RACE	56
*phot*2 R2	GACCAATGCTGCTCCAAGGACAA	5' RACE	56
*vde*1 F1	TACGAGCGAAGAAGCCAAGGAGATGT	3' RACE	56
*vde*1 R1	GACTACATATTCGGCGGAGGTGTTCG	5' RACE	56
*vde*2 F1	GAGCAGAGAAGCATTATCAG	3' RACE	56
*vde*2 R1	TGTATCCGAGAGCCTTGA	5' RACE	56
*vde*2 F2	AATTGGTTGCGATGCTGGAGAG	3' RACE	56
*vde*2 R2	TTGGCAAGGATAAGTGTCGTACT	5' RACE	56
zep R1	TTTCCCAGTGTCACGATTGCCAGC	Coding region amplification	56
*lhcx*1 qF1	AGGAGTCGCAAAGCCAGGGAAGAA	qRT-PCR	56
*lhcx*1 qR1	TCGCCCACAAAGAAGCCCAAAGC	qRT-PCR	56
*lhcx*2 qF2	AAGTTGAAGGAGATGCAGACGAAGGA	qRT-PCR	55
*lhcx*2 qR2	CAGGGTCTTGGGATTGGCGGAAA	qRT-PCR	55
*lhcx*3 qF2	CGCCTACCGATGCTGAGGAGATGA	qRT-PCR	55
*lhcx*3 qR2	CGTCCATTGTTGAGTTCCTTCGTCTG	qRT-PCR	55
*lhcx*4 qF2	AGCTGAAGCATGGTAGACTCGCAATG	qRT-PCR	58
*lhcx*4 qR2	AGGCTAAGATCGCTGGCACATCAATC	qRT-PCR	58
*lhcx*5 qF1	GAGTCGCAAAGCCAGGGAAGAATCT	qRT-PCR	58
*lhcx*5 qR1	GACAATGCTTCAACTCCGCCTCAC	qRT-PCR	58
*phot*2 qF2	ATGCGAATGAGTTGCGGATTGC	qRT-PCR	56
*phot*2 qR2	CCTCTTCAACATCGTCAACCTCCT	qRT-PCR	56

F: forward primer; R: reverse primer
