# Supplementary material for: Enhancement of Non-photochemical Quenching as an Adaptive Strategy under Phosphorus Deprivation in the Dinoflagellate Karlodinium veneficum
Source: Front Microbiol. 2017 Mar 15;8:404. doi: 10.3389/fmicb.2017.00404 (PMC5350143; doi:10.3389/fmicb.2017.00404)
Supplement: Supplementary file 3 [file Image_1.PDF]

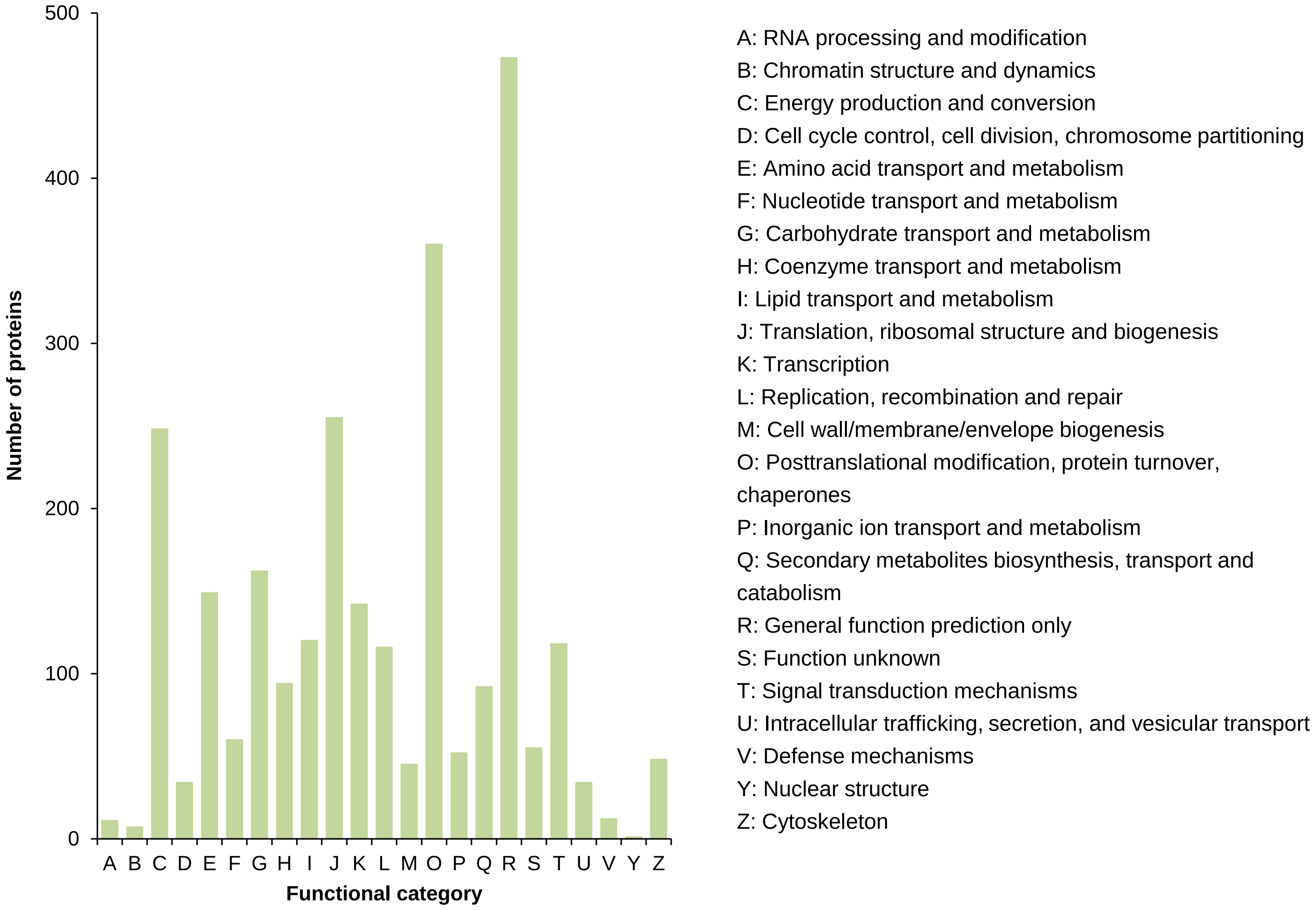

Supplementary Figure S1 Distribution of proteins identified in the iTRAQ proteomic analysis based on COG function description.
